# Ganaxolone enhances microglial clearance activity and promotes remyelination in focal demyelination in the *corpus callosum* of ovariectomized rats

**DOI:** 10.1111/cns.13195

**Published:** 2019-07-22

**Authors:** Abdeslam Mouihate, Samah Kalakh

**Affiliations:** ^1^ Department of Physiology, Faculty of Medicine Kuwait University Safat Kuwait

**Keywords:** electron microscopy, GABA_A_ receptor, g‐ratio, inflammatory cytokines, lysolecithin, microglia activation, myelin proteins

## Abstract

**Aim:**

Experimental studies have shown that the progesterone metabolite, allopregnanolone, is endowed with promyelinating effects. The mechanisms underlying these promyelinating effects are not well understood. Therefore, we explored the impact of allopregnanolone's synthetic analogue, ganaxolone, on remyelination and microglial activation following focal demyelination in the corpus callosum of ovariectomized rats.

**Methods:**

Ovariectomized adult Sprague Dawley rats received a stereotaxic injection of 2 µL of 1% lysolecithin solution in the *corpus callosum* followed by daily injections of either ganaxolone (intraperitoneal injection [i.p.], 2.5 mg/kg) or vehicle. The demyelination lesion was assessed 3 and 7 days postdemyelination insult using Luxol fast blue staining and transmission electron microscopy. The expression levels of myelin proteins (MBP, MAG, MOG, CNPase) were explored using Western blot. The inflammatory response and clearance of damaged myelin were evaluated using immunofluorescent staining (Iba1, dMBP, GFAP) and multiplex enzyme‐linked immunosorbent assay (IL‐1β, TNF‐α, IL‐4, IL‐10, IL‐6).

**Results:**

Systemic administration of ganaxolone promoted remyelination of lysolecithin‐induced demyelination, upregulated the expression of major myelin proteins, and enhanced microglial clearance of damaged myelin. Astrocytosis, as well as locally produced pro‐ and antiinflammatory cytokines, was not affected by ganaxolone treatment.

**Conclusion:**

Ganaxolone promotes remyelination in response to focal demyelination of the *corpus callosum* of ovariectomized rats. This effect is, at least in part, mediated by enhancing microglial clearance of myelin debris, which creates a conducive environment for a successful remyelination process.

## 1 INTRODUCTION

Several experimental studies suggest that allopregnanolone (ALLO), a metabolite of progesterone, is endowed with neuroprotective effects,^1^ likely due to its GABA_A_ receptor‐mediated antiinflammatory action.^2^ Brain maladies such as demyelination are associated with a local inflammatory response. There is an increased interest in the role of ALLO‐activated GABAergic system in de/remyelination processes. GABAergic tone was shown to be reduced in MS patient.^3^ Furthermore, postmortem studies showed reduced levels of ALLO in the white matter of MS patients.^4^ Experimentally, pharmacological activation of GABAergic signaling pathway(s) resulted in reduced paralysis in the animal model of multiple sclerosis, experimental autoimmune encephalomyelitis (EAE).^2^ However, our knowledge of the cellular and molecular mechanisms underlying the promyelinating effect of ALLO is limited.

It has been noted that MS symptoms are reduced during late pregnancy, when levels of ALLO are very high.^5^, ^6^, ^7^ There is a possibility that ALLO plays a major role in promoting remyelination during pregnancy.^8^ However, this promyelination effect could also be mediated by several other pregnancy‐associated steroid hormones. In this study, we aimed to investigate the specific effect of ALLO on de/remyelination processes away from the complex hormonal milieu associated with pregnancy. Hence, de/remyelination was explored in ovariectomized rats (OVX).

ALLO has a low bioavailability.^9^ Therefore, we explored the cellular and molecular effects of a synthetic analogue of ALLO, known as ganaxolone (GNX, 3α‐hydroxy‐3β‐methyl‐5α‐pregnan‐20‐one), on demyelination.^10^, ^11^ GNX is stable because the 3β‐methyl substituent in its chemical structure prevents its conversion to ALLO precursor.^12^ Similar to ALLO, GNX acts through GABA_A_ receptor (GABA_A_R) and increases the opening time of GABA_A_R to chloride ions.^10^, ^11^

The aim of this study was to investigate the effect of GNX on remyelination and the inflammatory response associated with demyelination, which involves the activation of microglia and astrocytes.^13^, ^14^ To avoid the systemic inflammatory response associated with EAE model, we explored the effect of GNX on lysolecithin‐induced focal demyelination of the *corpus callosum* of OVX rats. Lysolecithin is a detergent‐like gliotoxin that causes the dissolution of oligodendrocyte membranes causing their death and subsequent demyelination.^15^ Our data show that GNX enhances remyelination in the *corpus callosum* of OVX rats. It upregulates the expression of key myelin proteins, increases axonal myelination, and enhances clearance of damaged myelin by microglia.

## 2 METHODS

### 2.1 Animals

Female Sprague Dawley (SD) rats (230‐260 g) were obtained from the Animal Resources Centre, Faculty of Medicine, Kuwait University. Animals had access to pelleted chow and water ad libitum and were kept under a 12‐hour light/dark cycle (light 7 am‐7 pm). All experimental procedures were in line with the guidelines set by the Canadian Council on Animal Care and approved by the Kuwait University Health Science Center Animal Research Ethics Committee.

### 2.2 Ovariectomy surgery

Bilateral ovariectomy was performed on 6‐week‐old rats to minimize the effect of circulating hormones on the outcome of de/remyelination.^16^ Animals were anesthetized by an intraperitoneal injection of a mixture of ketamine (50 mg/kg) and xylazine (5 mg/kg), and both ovaries were surgically removed as previously described.^17^ Rats were returned to their home cages and left undisturbed for a period of 2 weeks.

### 2.3 Focal demyelination

Animals underwent stereotaxic surgery to induce a demyelination lesion in the *corpus callosum* 2 weeks post‐OVX as previously described.^18^ Briefly, a ten‐microliter Hamilton syringe (27 gauge; Hamilton Company) was stereotaxically positioned to deliver bilateral injection of 2 µL of lysolecithin solution (1% of lysolecithin in pyrogen‐free saline solution) into the *corpus callosum* at a rate of 1 µL/min using the following coordinates: bregma: 0 mm, anterolateral: ±2 mm, and dorsoventral: −3.3 mm.^19^ The syringe was left in place for four extra minutes to allow for the diffusion of the lysolecithin solution into the *corpus callosum*.

### 2.4 GNX treatment and tissue collection

GNX was dissolved in saline solution containing 10% of DMSO. OVX rats received once daily injections of GNX (i.p., 2.5 mg/kg) starting at 2 hours postfocal demyelination insult until the 3rd or the 7th day postsurgery. This GNX dose was chosen because it has been shown to moderately affect neuronal activity in experimental models of seizure.^20^ Control OVX rats received daily i.p. injections of an equivolume of saline solution containing 10% of DMSO (230‐260 µL). Brain tissues were collected at 3 and 7 days postdemyelination insult, to study the peak of inflammation and the initial phase of remyelination, respectively.^15^, ^21^ Rats were transcardially perfused with ice‐cold phosphate‐buffered saline (PBS) under urethane anesthesia (i.p., 1.5 mg/kg). For histological and immunofluorescence studies, brain tissues were postfixed with 10% neutral‐buffered formalin solution (Sigma‐Aldrich) for 48 hours and then embedded with paraffin (Sigma‐Aldrich).

Brain tissues were then processed for immunofluorescent detection of markers of microglia (ionized calcium binding adaptor molecule 1 or Iba1), astrocytes (glial fibrillary acidic protein or GFAP), and damaged myelin basic protein (dMBP) as previously described.^18^ The brain sections were incubated with primary antibodies overnight (see details in Table 1), then washed and incubated with either Alexa Fluor 488‐ or Alexa Fluor 555‐tagged secondary antibodies (1:1000, Invitrogen) for 2 hours at room temperature (see Table 1). Images of brain sections were acquired with a confocal microscope (Zeiss LSM 700 microscope) using either 40× or 63× objectives.

**Table 1:** Primary antibodies used in immunofluorescence and Western blot

Primary antibodies	Dilution	Company	Catalogue #	Secondary antibodies
Mouse monoclonal antibody anti‐MAG	1:1000	Abcam, Cambridge, MA, USA	ab89780	HRP‐conjugated donkey anti‐mouse IgG
Mouse monoclonal antibody anti‐MOG	1:1000	Millipore, MA, USA	MAB5680	HRP‐conjugated donkey anti‐mouse IgG
Mouse monoclonal antibody anti‐CNPase	1:2000	Sigma‐Aldrich, St. Louis, Mo., USA	C5922	HRP‐conjugated donkey anti‐mouse IgG
Mouse monoclonal antibody anti‐myelin basic protein	1:5000	Calbiochem, Billerica, MA, USA	NE1019	HRP‐conjugated donkey anti‐mouse IgG
Rabbit polyclonal antibody anti‐actin	1:5000	Sigma‐Aldrich, St. Louis, Mo., USA	A2066	HRP‐conjugated donkey anti‐rabbit IgG
Mouse monoclonal antibody anti‐iNOS	1:1000	BD Biosciences, San Jose, CA, USA	610432	HRP‐conjugated donkey anti‐mouse IgG
Mouse monoclonal antibody anti‐arginase‐1	1:1000	BD Biosciences, San Jose, CA, USA	610708	HRP‐conjugated donkey anti‐mouse IgG
Rabbit polyclonal antibody anti‐damaged myelin basic protein	1:1000	Millipore, MA, USA	AB5864	Alexa Fluor 488 donkey anti‐rabbit IgG
Goat polyclonal antibody anti‐Iba1	1:2000	Abcam, Cambridge, MA, USA	ab107159	Alexa Fluor 555 donkey anti‐goat IgG
Rabbit polyclonal antibody anti‐GFAP	1:5000	Sigma‐Aldrich, St. Louis, Mo., USA	G9269	Alexa Fluor 555 donkey anti‐rabbit IgG
Goat polyclonal antibody anti‐neurofilament	1:2000	Santa Cruz Biotechnology, Santa Cruz, CA, USA	sc‐16143	Alexa Fluor 488 donkey anti‐goat IgG
Mouse monoclonal antibody anti‐myelin basic protein	1:2000	Calbiochem, Billerica, MA, USA	NE1019	Alexa Fluor 555 donkey anti‐mouse IgG
Mouse monoclonal antibody anti‐GABA_A_Rγ2	1:200	Millipore, MA, USA	MABN875	Alexa Fluor 488 donkey anti‐mouse IgG

For transmission electron microscopy studies, the lesioned area of the *corpus callosum* was collected under a dissecting microscope, postfixed in 3% glutaraldehyde for 3 hours, and was embedded in epoxy resin embedding media. For Western blot experiments, the lesioned area of the *corpus callosum* was dissected, snap‐frozen in liquid nitrogen, and stored in −80°C until use as previously described.^14^

### 2.5 Histology

Paraffin‐embedded brains were sagittally cut at 5 μm thickness and mounted on SuperFrost Plus slides (SuperFrost^®^ Plus Micro Slide; VWR). Luxol fast blue (LFB) staining was performed to assess the extent of the demyelination lesion as described previously.^14^ The images of three brain sections from the center of the demyelination lesion of 4‐5 different rats per group were acquired and used for the image analysis. Images of the *corpus callosum* were acquired using an AxioVision software (Carl Zeiss). The demyelinated area was delineated and measured using ImageJ software (National Institute of Health) as previously described.^14^

### 2.6 Transmission electron microscopy

Epoxy resin embedded tissues were sectioned (0.5 μm) with a diamond knife on an ultramicrotome and mounted on slides. The sections were then stained with 1% toluidine blue and examined under Zeiss Axio Observer A1 microscope (Carl Zeiss) to confirm the presence of the lesion in the area collected. Ultrathin sections (100 nm) were then obtained and mounted on copper grids. Sections were stained with uranyl acetate and lead citrate. Images of the stained sections were acquired using 10 000× objective (JEOL's JEM‐1200 EXII Scanning Transmission Electron Microscope). Analysis was performed on three to six images of randomly selected areas from three different rats for each treatment group. A total of 954 axons with about 400 axons in each rat group were analyzed. The number of both demyelinated and myelinated axons was calculated using the ImageJ software. Each was then expressed as a percentage of the total number of axons in the observed field as previously described.^14^ In addition, the g‐ratio of myelinated axons was calculated by dividing the diameter of the axon without myelin sheath by the diameter of the axon with myelin sheath.^23^ This ratio is widely used as an index of axonal myelination.^24^

### 2.7 Western blot

The *corpus callosum* tissue was collected as described above and processed for Western blot as previously described.^14^, ^16^ Briefly, proteins were extracted and separated on an SDS‐PAGE gel. Subsequently, a semidry transfer was used to transfer the proteins into a nitrocellulose membrane. The membranes were then incubated overnight with primary antibodies directed against different myelin proteins (Table 1). The membranes were then rinsed and incubated with horseradish peroxidase (HRP)‐conjugated secondary antibodies for 2 hours at room temperature. Detection of the protein bands was performed using the enhanced chemiluminescent methodology (ECL Prime kit; GE Healthcare). The membranes were reused to detect the housekeeping protein, actin. Densitometric measurements were performed using ImageJ software.^22^ Optical density (OD) profile was determined for all protein bands. The area under the curve of the OD profile was used as an indicator of the quantity of protein. The optical density of each protein band was determined and expressed as a ratio of that of actin protein.

### 2.8 Immunofluorescence

Sagittal brain sections were cut at a thickness of 5 µm and mounted on SuperFrost Plus slides (VWR). The brain sections were processed for immunohistochemistry as previously described.^13^, ^18^ Brain sections were incubated with primary antibodies (Table 1) followed by secondary antibodies tagged with either Alexa Fluor 488 or Alexa Fluor 555 for multiple labeling. Cell nuclei were stained with the blue fluorescent 4′,6‐diamidino‐2‐phenylindole (DAPI; Electron Microscopy Sciences). Immunofluorescent images were acquired using a confocal microscope (Zeiss LSM 700 microscope; Carl Zeiss).

The local inflammatory response associated with demyelination was evaluated using the astrocyte marker GFAP and the microglia marker Iba1. This analysis was performed at the center of the lesion, where these cells showed the highest level of activation. For the analysis of GFAP, three randomly selected areas from each of the acquired images were used in the analysis. These images were obtained from 3‐4 brain sections derived from 4 to 5 different rats in each rat group. The immunoreactivity in these areas was binarized, and the fraction area occupied by GFAP immunoreactive cells was measured using a macro (Measure) in ImageJ software.^22^ The percentage of the total area occupied by GFAP immunoreactivity was used as an index of astrocytic activation (astrocytosis) as previously described.^13^ Alternatively, astrocytosis was evaluated by measuring the optical density of GFAP immunoreactivity. Microglial activation was assessed by quantifying the density of Iba1^+^ cells present at the center of the lesion. Microglial cells within the center of the lesion in 3‐4 brain sections, derived from 5 different rats in each rat group, were counted using the cell counter macro in ImageJ software 22 as previously described.^8^ Density of myelin debris in the lesioned area of the corpus callosum was evaluated using an antibody against dMBP. (SD1 in Appendix S1) illustrates the area in which assessment of local inflammation was carried out. All the above analyses were performed by an observer blind to the treatment received by each rat.

### 2.9 Multiplex enzyme‐linked immunosorbent assay

Activated microglia and astrocytes produce a range of inflammatory cytokines in the vicinity of the demyelination lesion. These inflammatory cytokines were measured using a multiplex Luminex assay (RECYTMAG‐60K; EMD Millipore Corporation), which allows the measurement of multiple cytokines simultaneously in a small amount of brain tissue (ie, the demyelinated area of the *corpus callosum*). The lesioned *corpus callosum* tissues were homogenized, and proteins were extracted for the multiplex ELISA. Each sample was assayed in duplicate and was tested for 3 proinflammatory (IL‐1 β, TNF‐α, and IL‐6) and 2 antiinflammatory cytokines (IL‐4 and IL‐10). The detection sensitivity of the assays was 2.8 pg/mL for IL‐1β, 1.9 pg/mL for TNF‐α, 3.1 pg/mL for IL‐4, 2.7 pg/mL for IL‐10, and 30.7 pg/mL for IL‐6. The inter‐assay variability was 11.3% for IL‐1β, 10.8% for TNF‐α, 10.7% for IL‐4, 9.0% for IL‐10, and 12.7% for IL‐6. The intra‐assay variability was 3.6% for IL‐1β, 2.7% for TNF‐α, 3.1% for IL‐4, 3.8% for IL‐10, and 2.3% for IL‐6. Cytokine concentrations were calculated using Luminex Manager Software (Luminex Software Inc).

### 2.10 Statistics

Data were compared using unpaired Student's *t* test. Statistical significance was declared when the *P* value was less than .05. ELISA results comparing 3 days vs 7 days postinjury were done using two‐way ANOVA followed by the Bonferroni post hoc test. All data and figures are presented as mean ± standard error (SEM).

## 3 RESULTS

### 3.1 GNX enhances remyelination following lysolecithin‐induced demyelination

**Figure 1:**
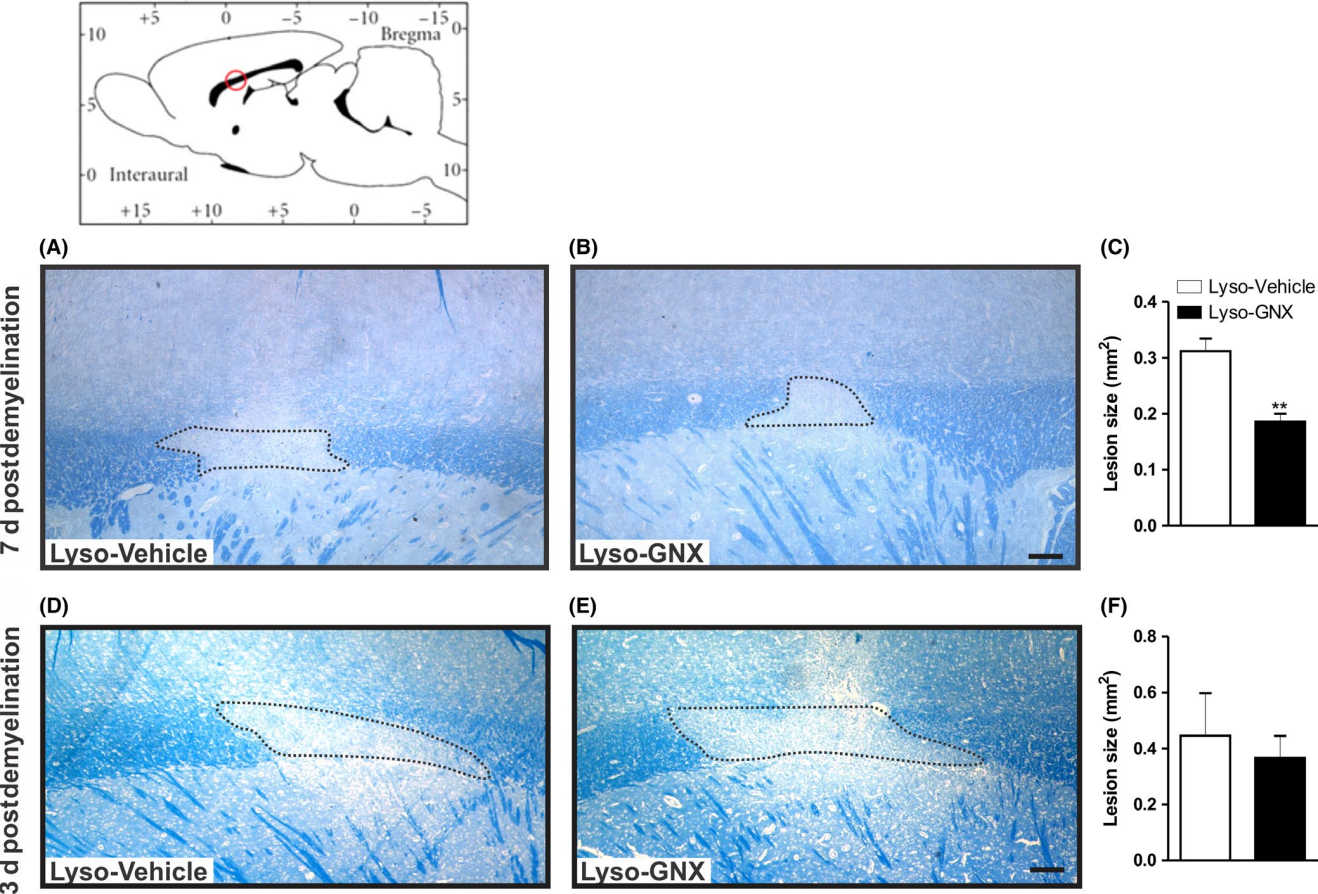
GNX reduces the demyelination lesion size 7 days post–lysolecithin‐induced demyelination in the *corpus callosum*. Brain diagram illustrates the approximate location of lysolecithin injection. Images of LFB staining in the lysolecithin‐injected *corpora callosa* of vehicle‐treated and GNX‐treated rats 7 days (A,B) and 3 days (D,E) postlesion. C, Bar graph showing that GNX significantly reduced the size of the demyelination lesion 7 days postlesion (Lyso‐GNX, n = 5 vs Lyso‐Vehicle, n = 5, *P* < .01). F, Bar graph shows that administration of GNX did not affect the size of the demyelination lesion when compared to vehicle‐treated rats 3 days postlesion (Lyso‐Vehicle: n = 5, Lyso‐GNX: n = 4, *P* > .05). Scale bar = 200 µm

Seven days postlysolecithin injection, the extent of myelin lesion was smaller in GNX‐injected when compared to that seen in control OVX rats given DMSO‐containing saline solution (Lyso‐Vehicle: n = 4, Lyso‐GNX: n = 5, *P* < .01; Figure 1A‐C). This DMSO‐containing saline solution does not significantly affect the size of the demyelination lesion when compared to that seen in rats given DMSO‐free saline (SD2 in Appendix S1).

To determine whether the reduced lesion size in GNX‐treated rats was due to enhanced remyelination or protection from demyelination injury, we measured the size of the lesion at 3 days postlysolecithin injection, a time point which corresponds to the peak of inflammation.^15^, ^21^ There was no difference in the size of demyelination lesion in the *corpus callosum* between vehicle‐treated and GNX‐treated rats 3 days postdemyelination (Lyso‐Vehicle: n = 5, Lyso‐GNX: n = 4, *P* > .05, Figure 1D‐F).

### 3.2 GNX promotes axonal myelination in the corpus callosum 7 days after lysolecithin‐induced demyelination

**Figure 2:**
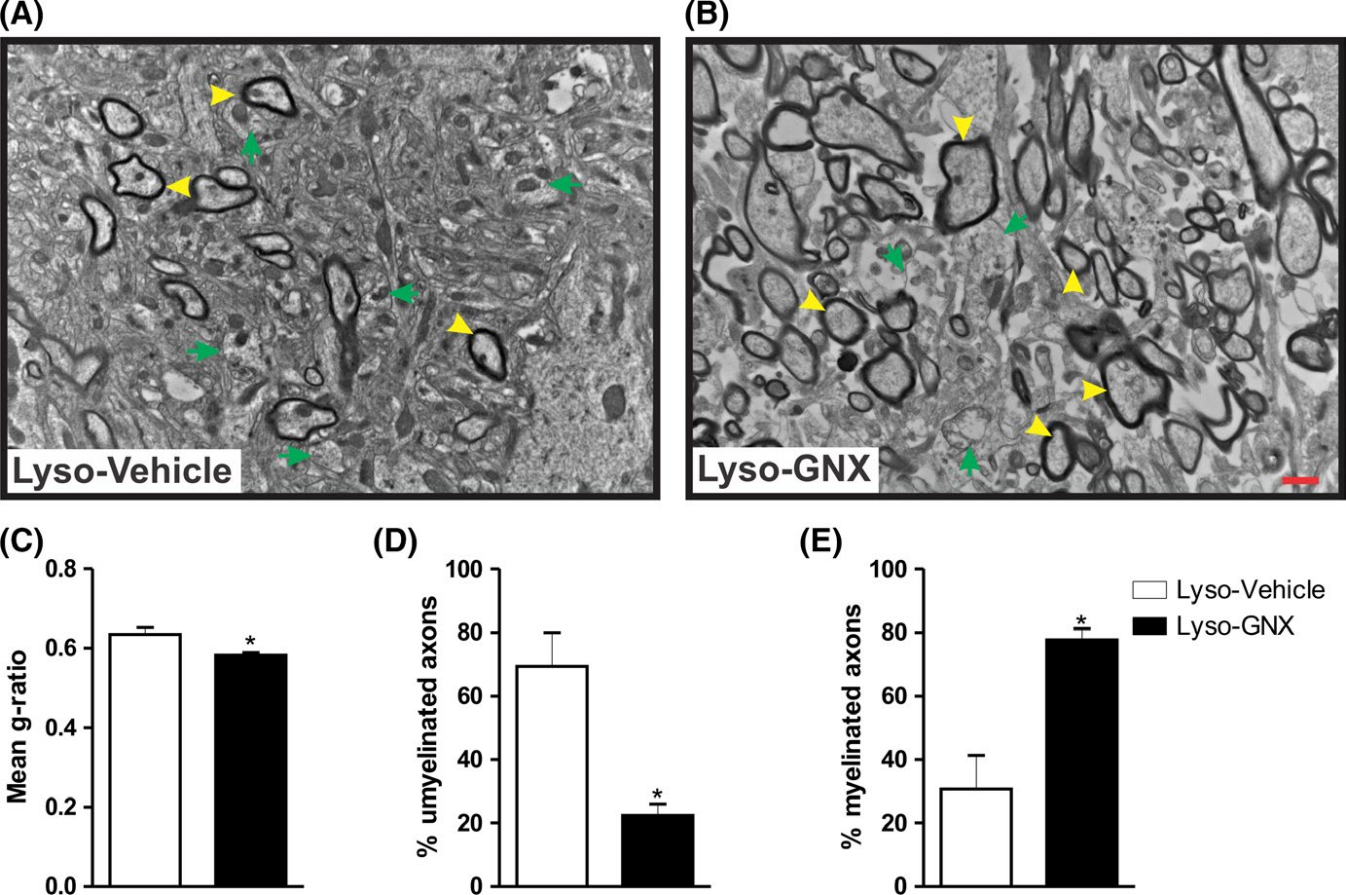
GNX enhances axonal myelination 7 days post–lysolecithin‐induced demyelination in the *corpus callosum*. (A) and (B) are TEM images of the demyelinated *corpora callosa* of vehicle‐treated and GNX‐treated rats, respectively. Yellow arrowheads indicate myelinated axons, while green arrows indicate unmyelinated ones. (C,D,E) Summary bar graphs showing that GNX treatment significantly reduced the g‐ratio compared to vehicle treatment (Lyso‐Vehicle: n = 3, Lyso‐GNX: n = 3, *P* < .05), resulted in a significantly lower number of unmyelinated axons compared to vehicle‐treated rats (*P* < .05), and significantly increased the number of myelinated axons compared to vehicle treatment (*P* < .05), respectively. Scale bar = 1 µm

The promyelinating effect of GNX was further assessed at the ultrastructural level using TEM (Figure 2A,B). A total of 954 axons were analyzed. GNX treatment significantly reduced the g‐ratio compared to vehicle‐treated rats (Lyso‐Vehicle: n = 3, Lyso‐GNX: n = 3, *P* < .05, Figure 2C). GNX treatment also led to a significant reduction in the percentage of unmyelinated axons (Figure 2D) and an increase in myelinated axons (Figure 2E) in the vicinity of the demyelination lesion.

### 3.3 GNX treatment enhances the expression of major myelin sheath proteins 7 days following lysolecithin‐induced demyelination

**Figure 3:**
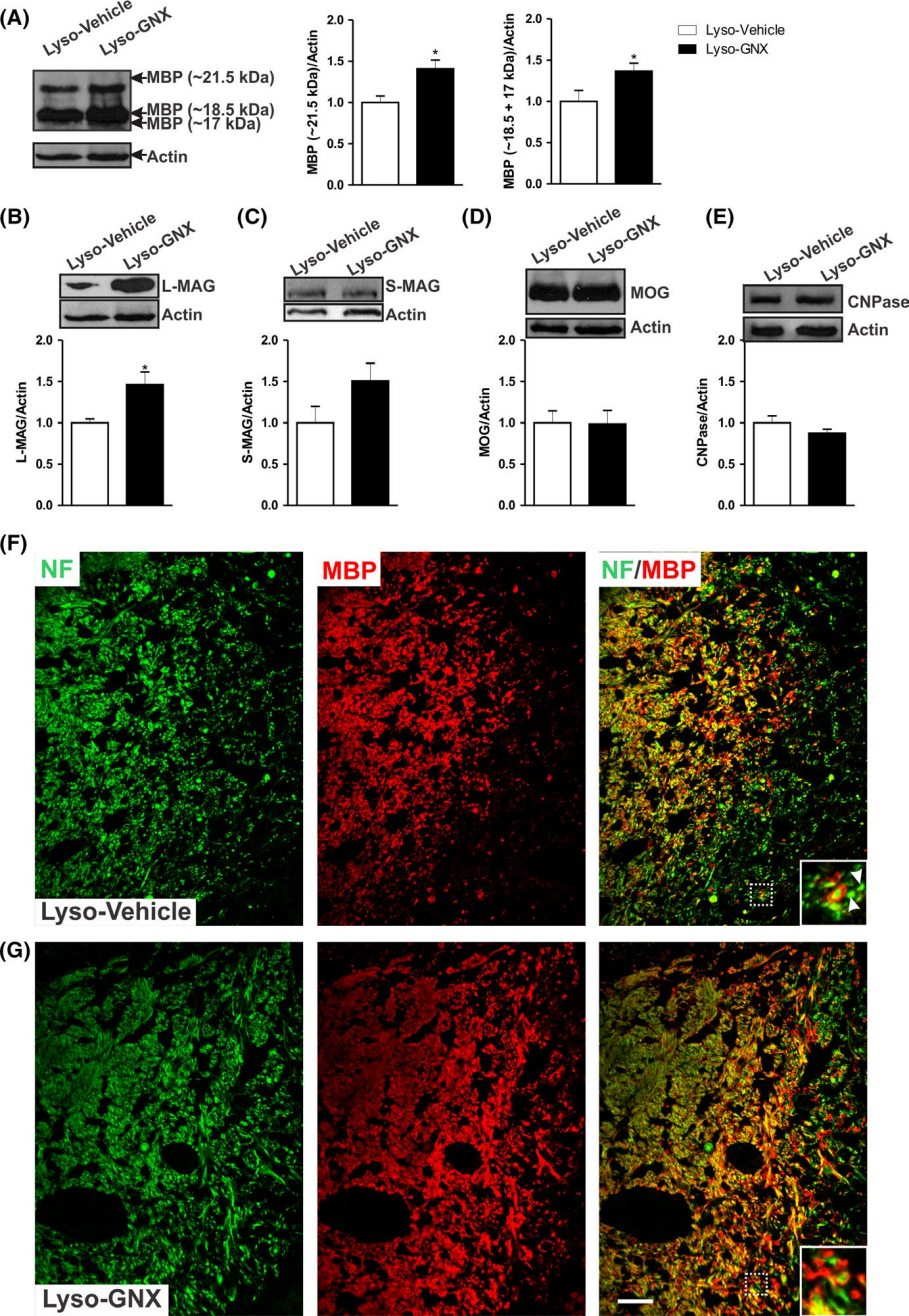
GNX promotes the expression of major myelin proteins 7 days post–lysolecithin‐induced demyelination in the *corpus callosum*. A, GNX treatment significantly increased the expression levels of the 21.5 kDa and the 18.5 + 17 kDa isoforms (Lyso‐Vehicle: n = 5, Lyso‐GNX: n = 4, *P* < .05). B, There was a significant increase in the expression of L‐MAG in GNX‐treated rats compared to vehicle‐treated rats (*P* < .05). Expression levels of S‐MAG (C), MOG (D), and CNPase (E) were not affected by GNX. (F) and (G) are immunofluorescent staining images of NF (green) and MBP (red) in the corpus callosum of vehicle‐treated and GNX‐treated OVX rats, respectively, 7 days postdemyelination. Inserts show higher magnifications of the area delimited by the white box. The axons (green) are surrounded by the myelin sheath (red). The arrowheads in (F) point to axons devoid of myelin. GNX treatment shows better axonal remyelination compared to vehicle‐treated rats. Scale bar = 50 µm

The myelination process involves an upregulation in a number of myelin proteins. We investigated the impact of GNX on the expression of major myelin proteins including MBP, MAG, MOG, and CNPase. Administration of GNX increased the expression of the 21.5 kDa and the 17 + 18.5 kDa MBP isoforms in the vicinity of demyelinated area of *corpus callosum* (Lyso‐Vehicle: n = 5, Lyso‐GNX: n = 4, *P* < .05, Figure 3A). GNX also significantly increased the expression of the large‐MAG isoform in the *corpus callosum* (*P* < .05, Figure 3B). GNX had no significant effect on the protein expression of either small‐MAG isoform (*P* > .05, Figure 3C), MOG (*P* > .05, Figure 3D), or CNPase (*P* > .05, Figure 3E). Remyelination at the edges of the lesion was also monitored using the axonal marker neurofilament (NF) and the myelin sheath marker myelin basic protein (MBP) 7 days postdemyelination insult. GNX treatment showed better axonal remyelination compared to vehicle‐treated rats (Figure 3F,G).

### 3.4 GNX reduces the density of microglia and myelin debris in the vicinity of the demyelination lesion 7 days following lysolecithin‐induced demyelination

**Figure 4:**
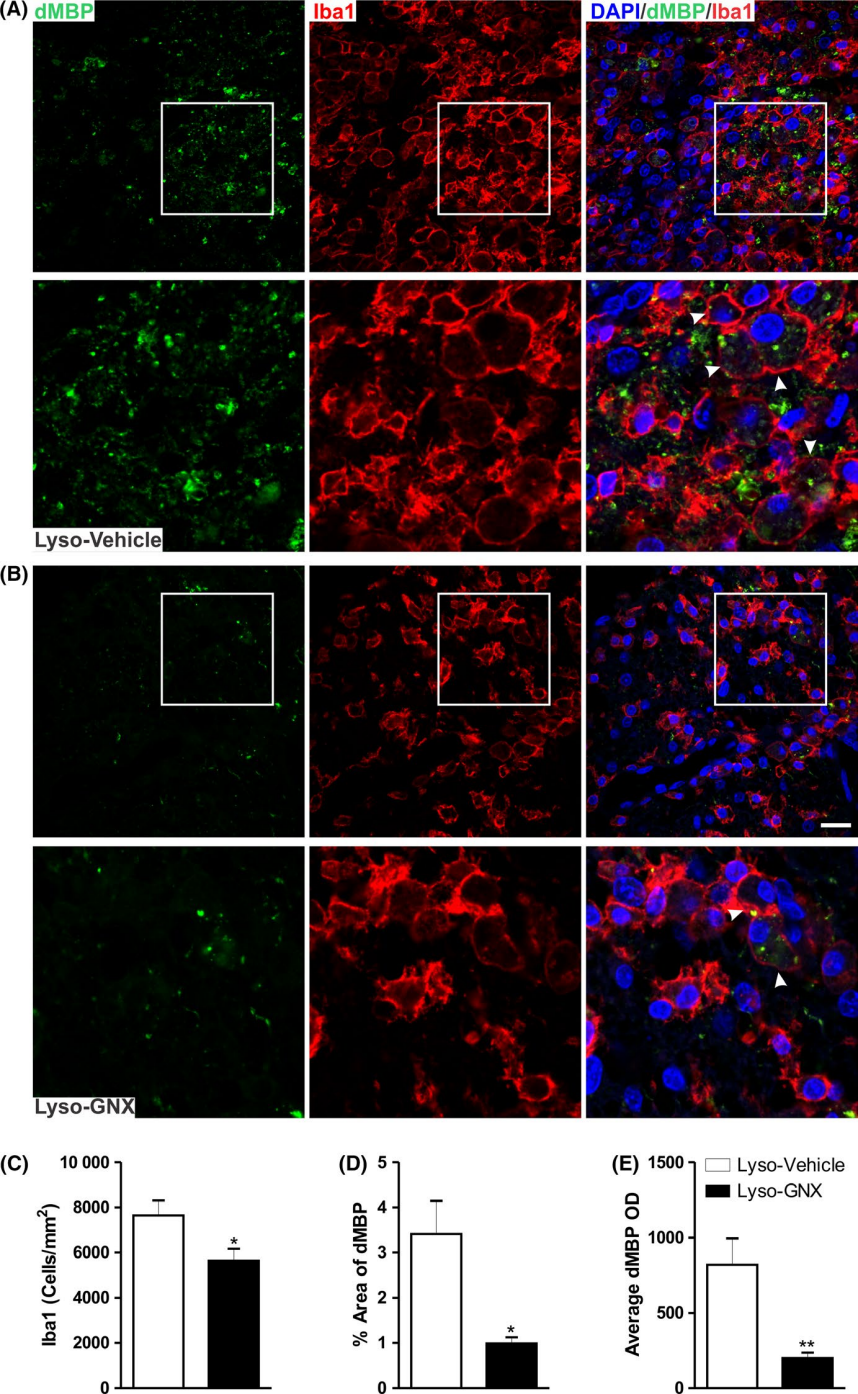
GNX reduced microglial cell density and enhanced clearance of myelin debris in the vicinity of the demyelination lesion. (A) and (B) show immunofluorescent staining image of damaged myelin basic protein (dMBP; green) and the microglial marker Iba1 (red) in vehicle‐treated and GNX‐treated rats, respectively. Arrowheads indicate microglia engulfing myelin debris. C, GNX significantly reduced the density of Iba1^+^ cells in the vicinity of the demyelination lesion compared to vehicle treatment (*P* < .05). The fraction area covered by dMBP (D) and the average OD of dMBP (E) were significantly reduced by GNX treatment compared to vehicle animals (*P* < .05 and *P* < .01, respectively). Scale bar = 50 µm

Lysolecithin‐induced demyelination is accompanied by a local inflammatory response. This response involves the activation of astrocytes and microglia within and around the demyelination lesion. In our hands, saline injection into the corpus callosum induced a moderate microglial activation. However, this microglial activation was negligible when compared to that seen in lysolecithin‐injected corpus callosum (SD3 in Appendix S1). Furthermore, lysolecithin injection results in highly activated microglia at the center of the lesion (Figure 4A,B and Figure SD1 in Appendix S1). This activation process is manifested by increased density of microglia, which show ameboid shape and retracted processes.^18^

Interestingly, GNX treatment significantly reduced the density of microglia (Iba1^+^ cells) at the center of the demyelination lesion (Lyso‐Vehicle: n = 5, Lyso‐GNX: n = 5, *P* < .05, Figure 4C). To assess the potential role of microglia in the clearance of damaged MBP (dMBP), microglial cells were colabeled with a marker specific to dMBP. The immunoreactivity to dMPB was seen within microglial cells. Importantly, daily injection of GNX for 7 days led to a significant reduction in dMBP (Figure 4D,E). This GNX‐induced reduction in dMBP was not seen at 3 days after the demyelination insult. Indeed, neither the density of Iba1^+^ cells nor the levels of dMBP were affected by GNX at 3 days postdemyelination insult (Lyso‐Vehicle: n = 4, Lyso‐GNX: n = 4, *P* > .05, SD4 in Appendix S1).

**Figure 5:**
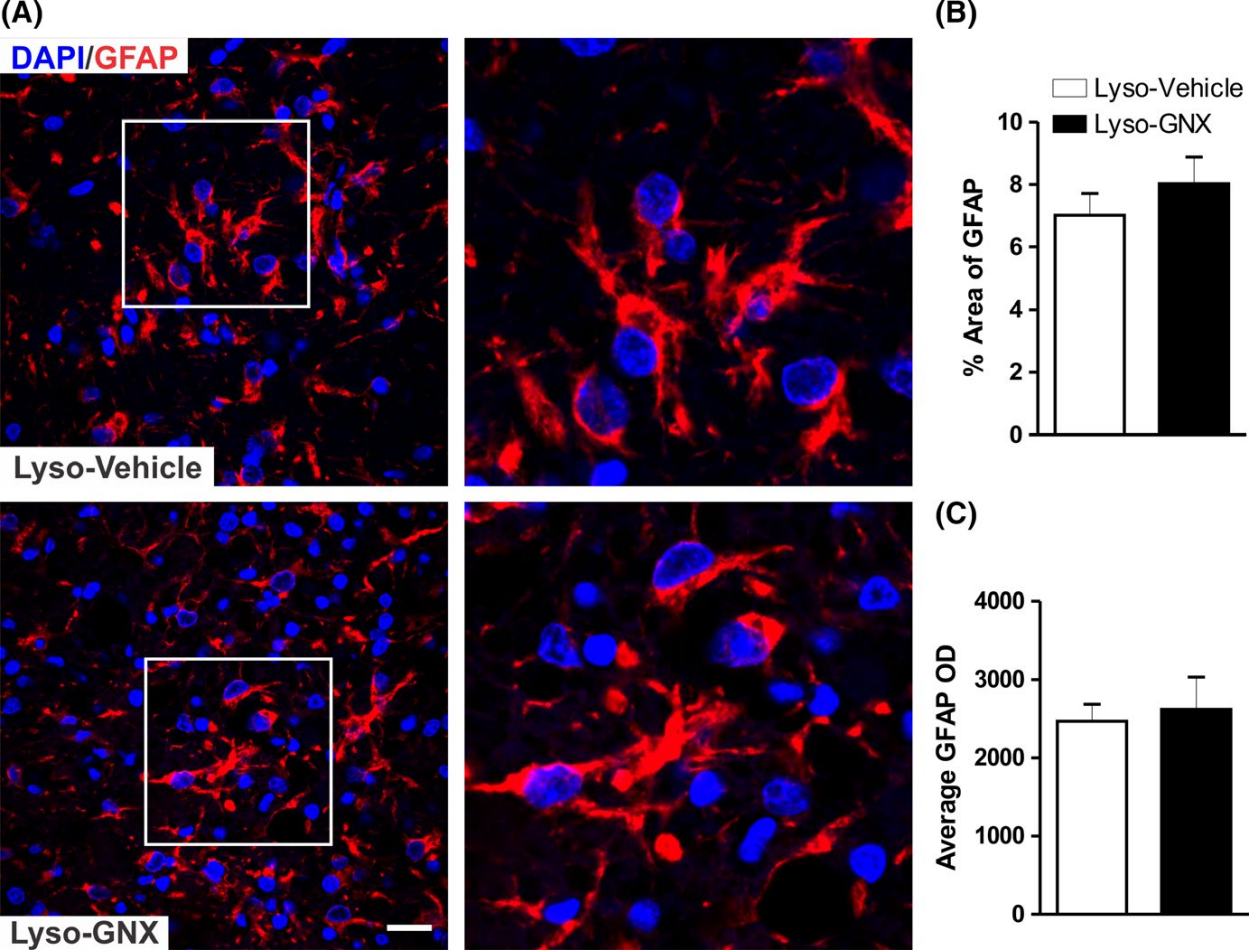
GNX does not affect astrocytic activation in the vicinity of the demyelination lesion. A, Immunofluorescent staining image of the astrocytic marker GFAP. GNX did not affect the percentage area covered by GFAP (B) or average GFAP optical density (C) in the vicinity of demyelinated area of the *corpus callosum* of OVX rats when compared to those seen in vehicle‐treated OVX rats (Lyso‐Vehicle: n = 5, Lyso‐GNX: n = 4, *P* > .05). Scale bar = 50 µm

In addition to microglial activation, demyelination also induces astrocytosis. In contrast to its effect on microglia, GNX did not significantly affect astrocytic activation in the vicinity of demyelinated area of the *corpus callosum* either at 7 days (Lyso‐Vehicle: n = 5, Lyso‐GNX: n = 4, *P* > .05, Figure 5) or at 3 days postdemyelination insult (Lyso‐Vehicle: n = 5, Lyso‐GNX: n = 4, *P* > .05, SD5 in Appendix S1).

We also evaluated the effect of GNX on microglial polarization by assessing M1 and M2 markers inducible nitric oxide synthase (iNOS) and arginase‐1, respectively. There was no significant effect of GNX treatment on microglial polarization at either 3 days (Lyso‐Vehicle: n = 5, Lyso‐GNX: n = 4, *P* > .05, SD6 A, B in Appendix S1) or 7 days postdemyelination insult (Lyso‐Vehicle: n = 7, Lyso‐GNX: n = 7, *P* > .05, SD6 C, D in Appendix S1). Furthermore, GNX also did not significantly affect the expression levels of pro‐ and antiinflammatory cytokines (IL‐1β, TNF‐α, IL‐6, IL‐4, and IL‐10) within the demyelination lesion at either 3 days or 7 days postdemyelination insult (SD7 in Appendix S1).

## 4 DISCUSSION

In the current study, we show that systemic administration of GNX, the synthetic analogue of ALLO, enhances remyelination following a focal demyelination lesion in the *corpus callosum* of OVX rats. This enhanced remyelination is manifested by an overall reduction in the size of demyelination lesion, which improved axonal myelination and an upregulation of multiple myelin proteins. These promyelinating properties were associated with enhanced microglia‐driven clearance of damaged myelin.

Experimental and clinical studies have pointed to the reduction of demyelination‐associated symptoms in MS patients during late pregnancy,^25^, ^26^, ^27^ likely through the action of ovarian hormones. However, the complexity of the hormonal milieu during pregnancy precludes the separation of the major promyelinating factors during pregnancy. Thus, we elected to explore the potential myelinating effect of one major pregnancy‐associated hormone on demyelination in OVX rats. Indeed, ALLO has been shown to increase drastically during late phase of pregnancy and could promote myelination.^8^ In the present study, we show that systemic administration of GNX, the synthetic analogue of ALLO, enhances remyelination following a focal demyelination lesion in the *corpus callosum* of OVX rats.

Microglial cells have been associated with a deleterious effect on neural tissues in brain‐related diseases. However, these immune‐competent cells facilitate the naturally occurring remyelination, as their depletion compromises recovery from demyelination insult.^28^, ^29^ Furthermore, the clearance of damaged MBP has been shown to promote remyelination in a model of cuprizone‐induced demyelination.^30^ In the present study, we show that systemic administration of GNX increases the clearance of damaged myelin at the demyelination lesion. These damaged myelin proteins were engulfed by microglial cells. GNX likely acts indirectly on activated microglia to enhance remyelination as these immune‐competent cells express GABA_A_R 31 (also see SD8 in Appendix S1), the main binding site of ALLO/GNX. Interestingly, the enhanced myelin debris clearance was associated with increased remyelination.

In addition to microglia, astrocytes also express GABA_A_R and could be a target of GNX. Our study showed that while demyelination induced astrocytosis, this astrocytosis was not significantly affected by GNX. Thus, it appears that GNX effect is mainly driven by microglia. The mechanism underlying this differential effect of ALLO on microglia and astrocytes is not clear. Previous studies have suggested that the modulation of GABA_A_R by ALLO is influenced by the receptor's subunit composition. Indeed, GABA_A_R containing γ2 subunit (GABA_A_R γ2) shows more sensitivity to the action of ALLO.^32^ The fact that the expression of GABA_A_R γ2 was mostly observed in microglia and to a lesser extent in astrocytes (see data 8 in Appendix S1) suggests that microglia are likely the preferred target of GNX. In addition to clearance of myelin debris, microglia can be polarized into either a proinflammatory M1 or regulatory M2 types. The M2 microglia have been shown to release a set of regulatory factors involved in the maturation of oligodendrocyte precursor cells.^33^, ^34^, ^35^, ^36^ In our hand, GNX‐induced enhanced clearance activity of microglia was dissociated from microglial polarization. Indeed, GNX treatment had no significant effect on the expression levels of major M1 (proinflammatory cytokines and iNOS) or M2 markers (antiinflammatory cytokines and Arg‐1) within the area of the demyelination lesion (SD6 and SD7 in Appendix S1). It seems that GNX affects intrinsic phagocytic action of microglia without significantly altering their polarization and the consequent production of inflammatory cytokines. This observed dissociation between phagocytic and inflammatory functions of microglia is in line with a previous study.^37^

Alternatively, GNX could promote myelination by a direct action on oligodendrocytes as these cells also express GABA_A_R.^38^, ^39^, ^40^, ^41^, ^42^ However, there are conflicting data as to whether direct activation of GABA_A_R affects OPC development.^43^, ^44^ In vitro evidence suggests that activation of GABA_A_R could promote myelination upon oligodendrocyte‐axon interaction.^45^ Activation of GABA_A_R in OPCs could lead to the migration of OPCs toward the demyelination lesion 46 to differentiate into mature oligodendrocytes.^47^ In line with these observations, we show that administration of GABA_A_R agonist led to an enhanced myelination manifested by increased myelin proteins and axonal myelination. It is plausible that GNX had a double‐pronged effects by promoting OPC migration and differentiation and enhancing the clearance capacity of microglia. The clearance of myelin debris is essential for the optimal myelin recovery.^30^

Maturing oligodendrocytes produce a series of myelin proteins, which are essential for myelin stability and function.^48^ In the present study, we show that GNX enhanced the expression levels of key myelin proteins including several MBP isoforms and the large MAG (L‐MAG). This stimulatory effect of GNX on myelin proteins led to enhanced number of myelinated axons. We have observed a peculiar effect of GNX on MAG isoforms, while it increased the expression levels of L‐MAG isoform; it had shown no significant effect on the expression levels of smaller isoform (S‐MAG). It is unclear how GNX differentially affects the expression of MAG isoforms. It has been shown that L‐MAG is the predominant isoform during early stages of brain development while the S‐MAG appears at later stages.^49^ Perhaps this developmental sequence of L‐MAG and S‐Mag is recapitulated during remyelination in adulthood. Functionally, the L‐MAG isoform plays a major role in the myelination process as L‐MAG–deficient mice show the same pathological abnormalities observed in total MAG‐deficient mice in CNS myelin.^50^, ^51^, ^52^

Ganaxolone presents an opportunity for managing the deleterious effect of local brain inflammation associated with several neurodegenerative diseases. This drug was tested in clinical trials for epilepsy and has been shown to be safe and tolerable.^53^, ^54^, ^55^, ^56^ The US Food and Drug Administration (FDA) has approved Orphan Drug Designation to GNX for the treatment of status epilepticus and fragile X syndrome (see http://www.fda.gov). To date, however, there are no clinical trials to evaluate the efficacy of GNX on demyelinating conditions such as MS. This experimental study gives a rationale for assessing the potential clinical use of GNX in demyelinating diseases.

## 5 CONCLUSION

We provided experimental evidence to support the promyelinating effect of GNX following demyelination. This promyelinating effect was observed at the ultrastructural, molecular, and cellular levels. Our experimental data suggest that GNX acts largely by enhancing clearance of myelin debris by microglia. This enhanced clearance might have led to a local environment conducive for the naturally occurring remyelination.

## CONFLICT OF INTEREST

The authors declare no conflict of interest.
