# Supplementary material for: Ganaxolone enhances microglial clearance activity and promotes remyelination in focal demyelination in the corpus callosum of ovariectomized rats
Source: CNS Neurosci Ther. 2019 Jul 22;26(2):240–50. doi: 10.1111/cns.13195 (PMC6978248; doi:10.1111/cns.13195)
Supplement: Supplementary file 1 [file CNS-26-240-s001.pdf]

Supplementary data

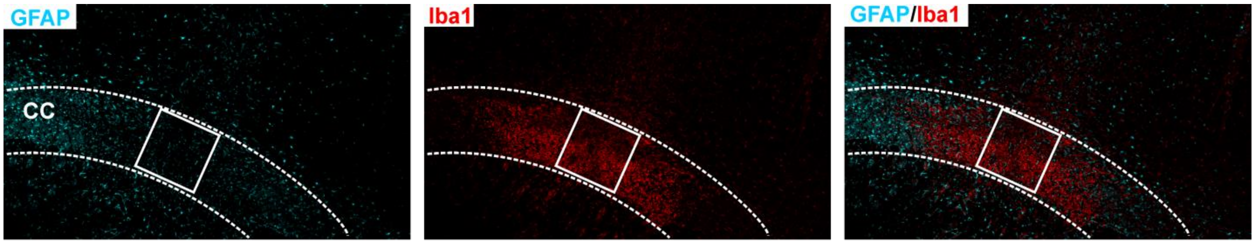

Supplementary data 1

Supplementary data 1: Lesion overview. Immunofluorescent images of the astrocyte marker (GFAP; cyan) and the microglial marker Iba1 (red). Microglia are highly activated at the center of the lesion, while activated astrocytes are mostly at the edges of the lesion. White box indicates the area at which density analysis of microglia and astrocytes was performed. CC: corpus callosum.

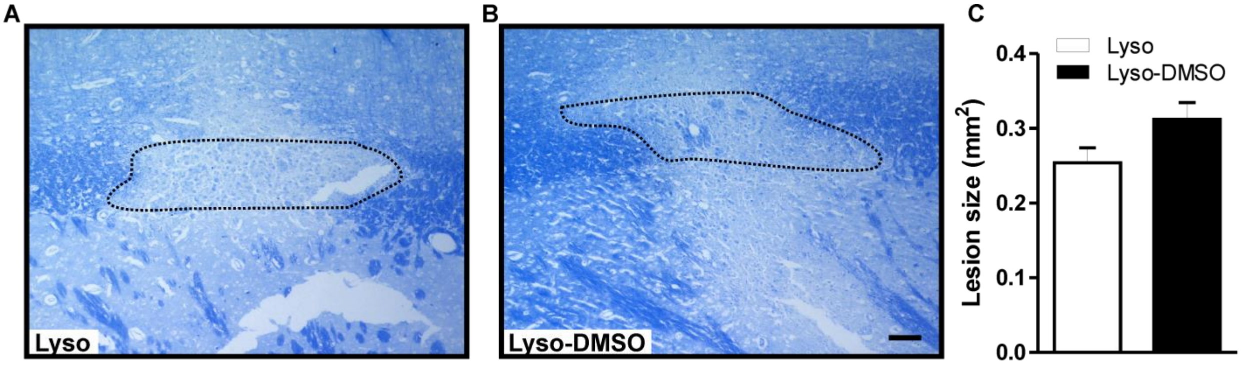

Supplementary data 2

Supplementary data 2: DMSO does not affect the size of the demyelination lesion. Images of LFB staining in the lysolecithin-injected *corpora callosa* with **(B)** or without **(A)** intraperitoneal injection of DMSO, 7 days post-demyelination insukt. **C)** Bar graph shows that DMSO injections did not affect the size of lysolecithin-induced demyelination lesion (Lyso: n=4, Lyso-DMSO: n=4, $p>0.05$). Scale bar = 200µm.

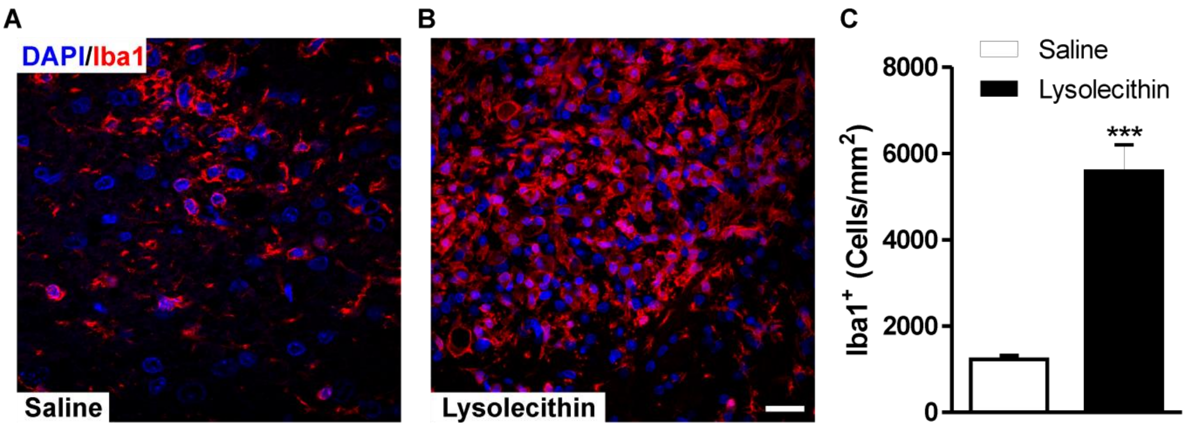

Supplementary data 3

Supplementary data 3: Impact of saline vs. lysolecithin on microglia at the site of injection in the corpus callosum. Immunofluorescent images of the microglial marker Iba1 (red) in the corpus callosum in saline- **(A)** and lysolecithin-injected female rats **(B)**, seven days after injection. Microglial activation following lysolecithin injection was significantly larger when compared to that seen in saline-injected animals (graph bar in **(C)**) (Saline: n=4, Lysolecithin: n=4, $p<0.001$)

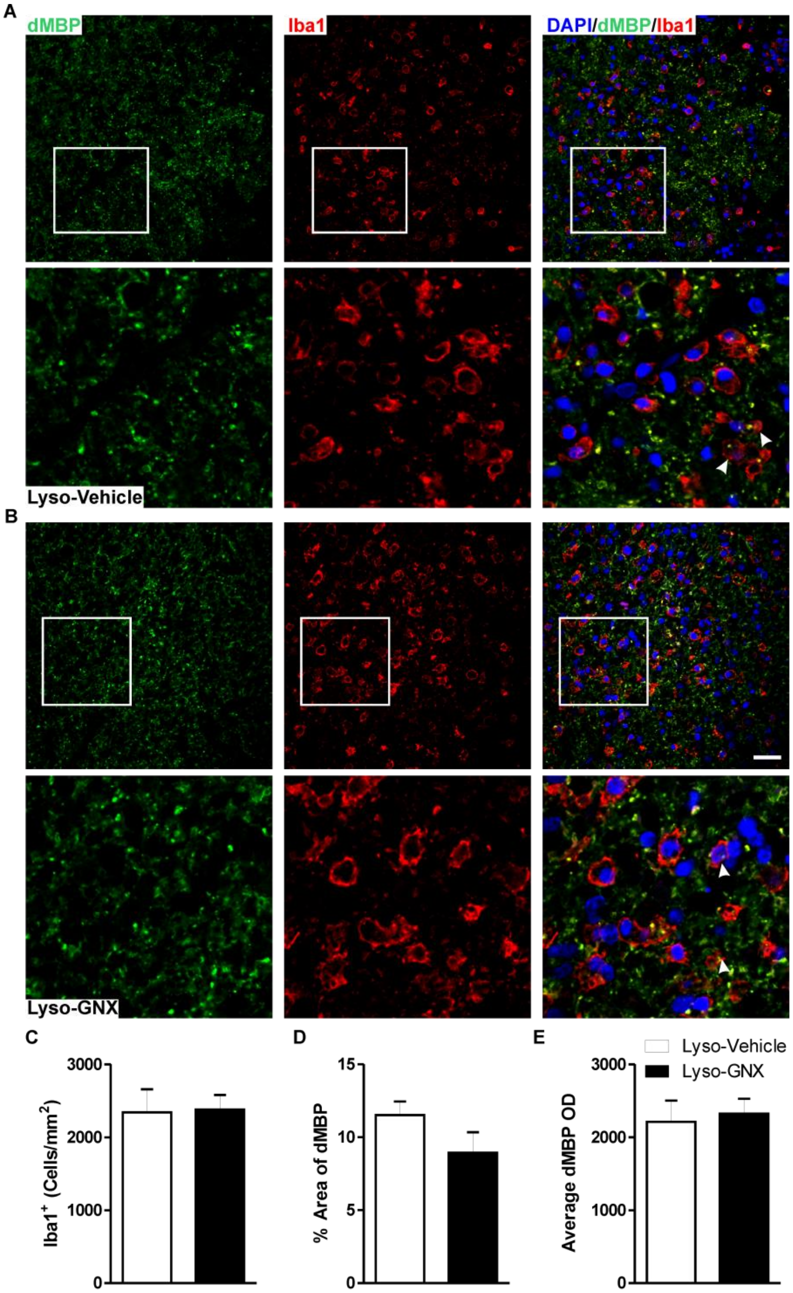

Supplementary data 4

Supplementary data 4: GNX does not affect microglial cell density or clearance of myelin debris in the vicinity of the lesion 3 days post-demyelination in OVX rats. Micrographs in **(A)** and **(B)** show immunofluorescent staining of damaged myelin basic protein (dMBP; green), the microglial marker Iba1 (red) and the nuclear marker DAPI (blue) in vehicle-treated (Lyso-Vehicle) and GNX-treated rats (Lyso-GNX) 3 days post-demyelination insult. White squares delimit areas shown in higher magnification below each micrograph. GNX did not significantly alter the density of Iba1^{+} cells **(C)**, the fraction area covered by dMBP **(D)**, or the average optical density (OD) of dMBP **(E)** in the vicinity of the demyelination lesion at this time point (p>0.05). Scale bar = 50 µm.

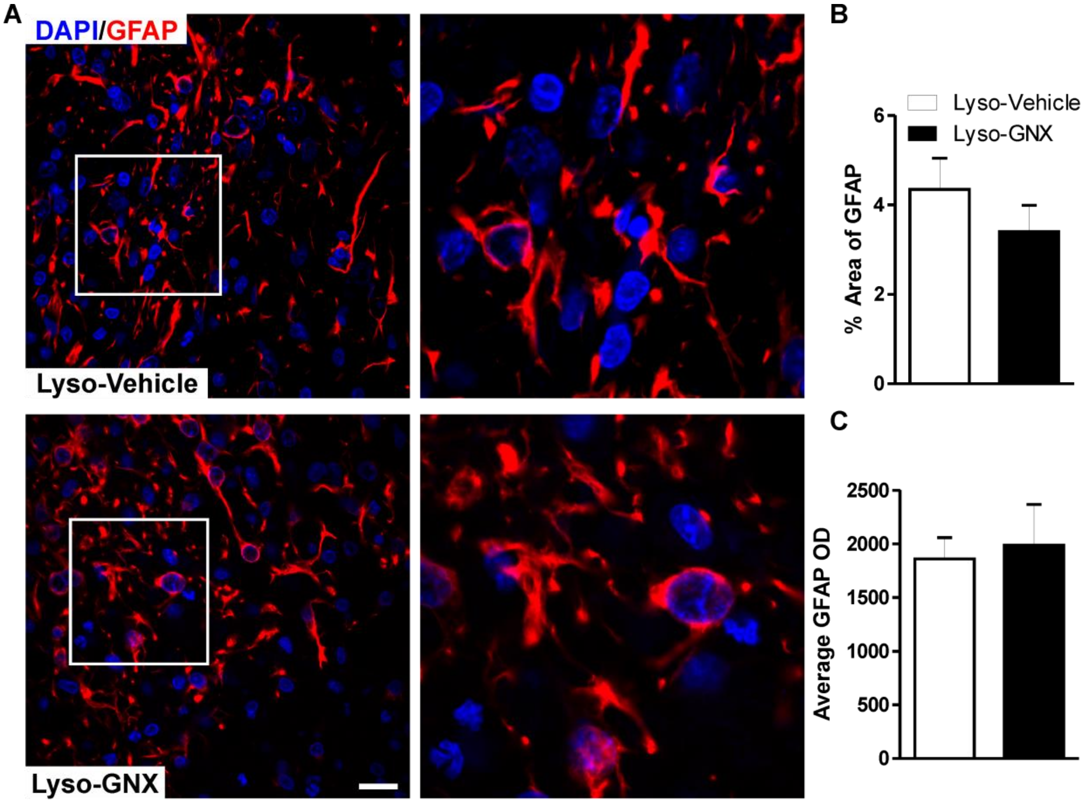

Supplementary data 5

Supplementary data 5: GNX does not affect astrocytic activation in the vicinity of the demyelination lesion 3 days post-lesion in OVX rats. (A) Immunofluorescent staining images of the astrocytic marker GFAP (red) and the nuclear marker DAPI (blue). GNX did not affect the percentage area covered by GFAP **(B)** or the average optical density (OD) of GFAP **(C)** in the vicinity of demyelinated area 3 days post-demyelination. White squares delimit areas shown in higher magnification on the right side of each micrograph. (Lyso-Vehicle: n=5, Lyso-GNX: n=4, $p>0.05$). Scale bar = 50 µm.

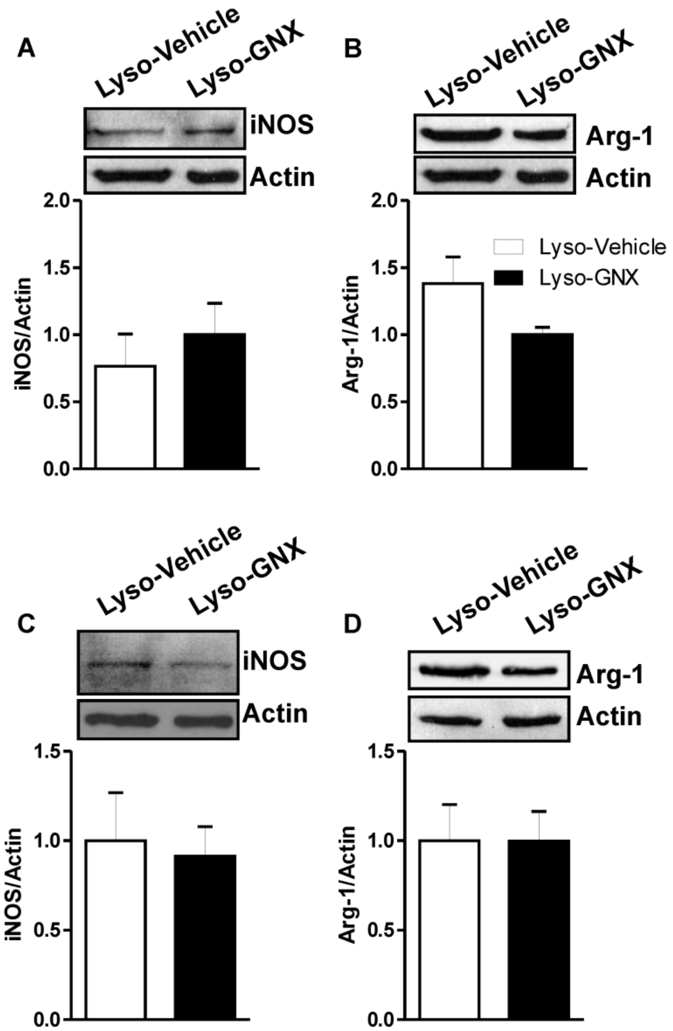

Supplementary data 6

Supplementary data 6: GNX does not affect microglial polarization within the demyelination lesion in OVX rats. Microglial activation markers were investigated using western blot at both 3 days (**A** and **B**) and 7 days post-demyelination insult (**C** and **D**). Classically activated (M1 type) and alternatively activated microglia (M2 type) were assessed using iNOS **(**M1 type, **A** and **C)** and Arg-1 (M2 type, **B** and **D**) markers. Intraperitoneal administration of GNX to OVX rats did not significantly affect the expression levels of iNOS or Arg-1 at either 3 days (Lyso-Vehicle: n=5, Lyso-GNX: n=4, $p>0.05$) or 7 days post-demyelination insult (Lyso-Vehicle: n=7, Lyso-GNX: n=7, $p>0.05$).

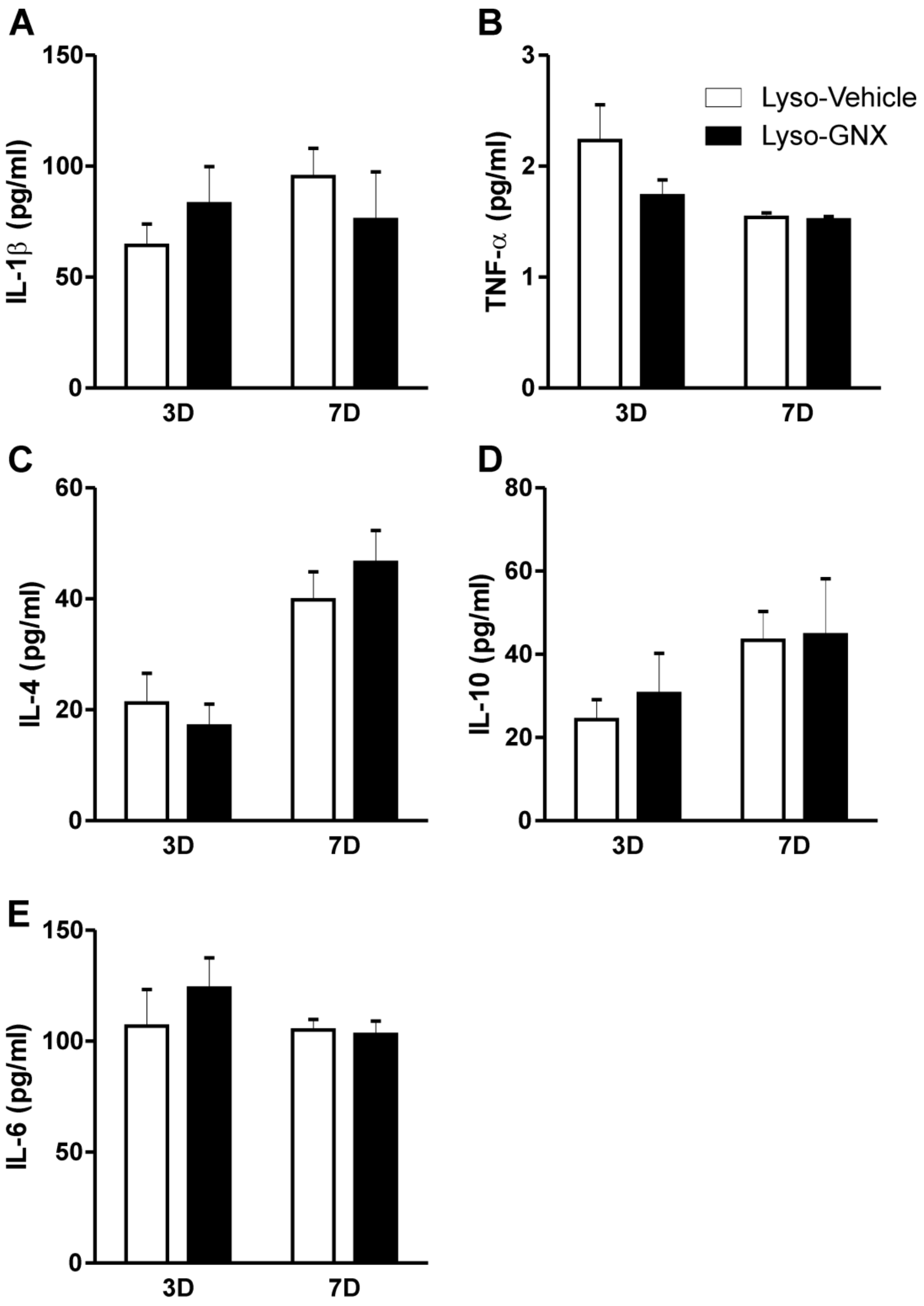

Supplementary data 7

Supplementary data 7: GNX does not affect the expression levels of pro- and anti-inflammatory cytokines in the demyelinated corpus callosum of OVX rats. The bar graphs show the levels of IL-1β **(A)**, TNF-α **(B)**, IL-4 **(C)**, IL-10 **(D)** and IL-6 **(E)** in the demyelinated corpus callosum of vehicle-treated (Lyso-Vehicle) and GNX-treated (Lyso-GNX) OVX rats 3 days (3D) and 7 days (7D) post-demyelination insult [3D (Lyso-Vehicle: n=5, Lyso-GNX: n=5), 7D Lyso-Vehicle: n=7, Lyso-GNX: n=7)]. Intraperitoneal administration of GNX to OVX rats did not significantly affect the expression levels of IL-1β, TNF-α, IL-4, IL-10 or IL-6 within the demyelination lesion of the corpus callosum at either 3 or 7 days post-demyelination insult. Results are represented as mean ± SEM, $p>0.05$.

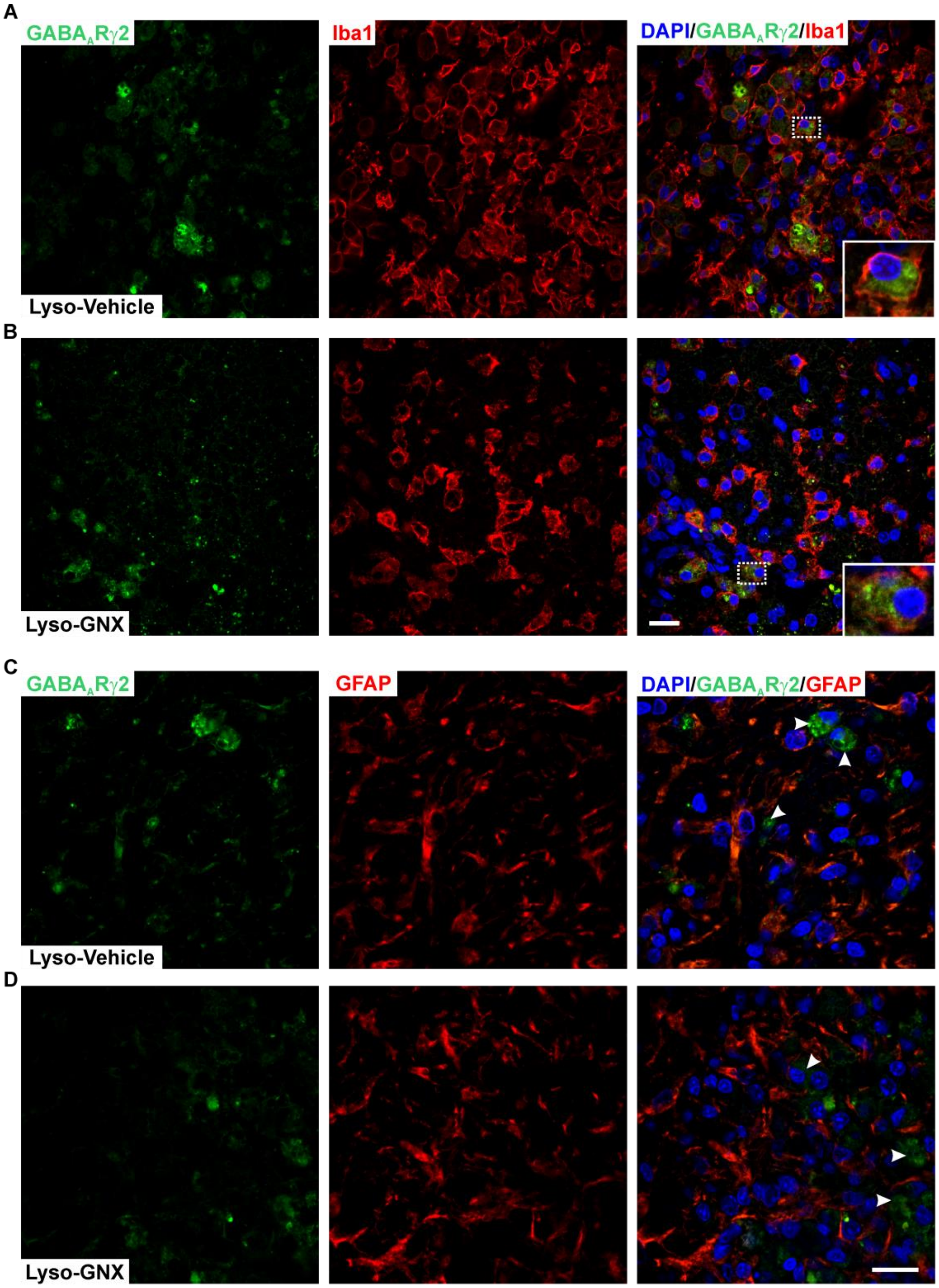

Supplementary data 8

Supplementary data 8: Expression of $GABA_AR\gamma2$ in microglia and astrocytes in the vicinity of the demyelination lesion. Co-immunfluorescent staining of $GABA_AR\gamma2$ (green) with either microglial (Iba1, red in middle panels in **A** and **B**) or astrocytic markers (GFAP, red in middle panels in **C** and **D**) in the vicinity of demyelinated corpus callosum of OVX rats given systemic injections of either vehicle (Lyso-Vehicle) or GNX (Lyso-GNX). The far-right micrograph panels show merged red and green channels and the nuclear staining with DAPI (blue). A large proportion of $Iba1^+$ cells co-expressed $GABA_AR\gamma2$. Insets in **(A)** and **(B)** illustrate magnified views of microglial cells expressing $GABA_AR\gamma2$. The arrowheads in **(C)** and **(D)** show that the $GABA_AR\gamma2$ was seen outside $GFAP^+$ cells. Scale bar = 50 μm.
